# Supplementary material for: Reduction of LPAR1 Expression in Neuroblastoma Promotes Tumor Cell Migration
Source: Cancers (Basel). 2022 Jul 9;14(14):3346. doi: 10.3390/cancers14143346 (PMC9322936; doi:10.3390/cancers14143346)
Supplement: Supplementary file 1 [file cancers-14-03346-s001.zip › Supplementary Figures.pdf]

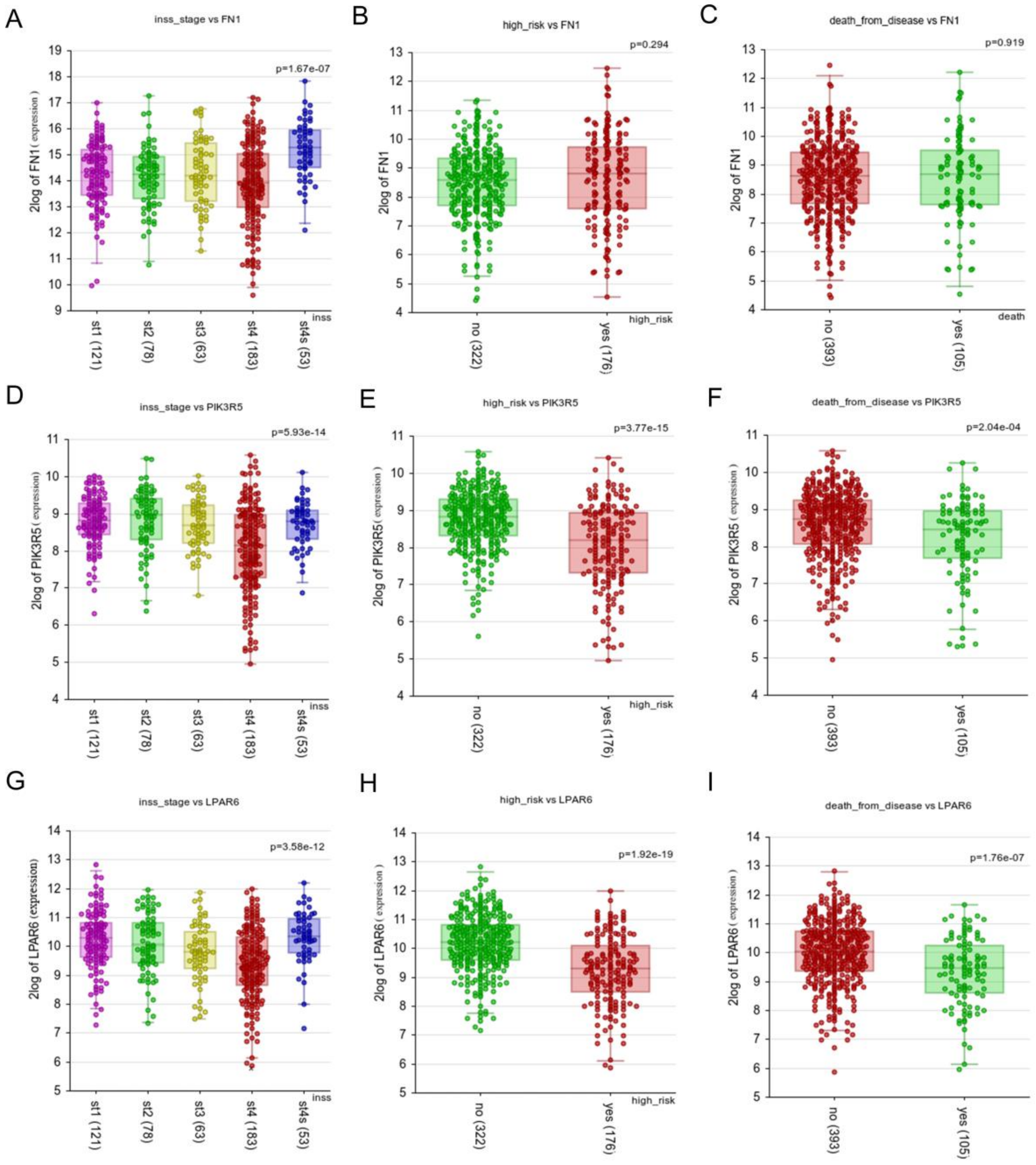

Figure S1. The expression pattern of FN1, PIK3R5 and LPAR6 in NB. The R2 database view-a-gene was used to analyze the expression pattern of screened genes based on the average mRNA expression of the 498 NB SEQC dataset, with $p < 0.05$ regarded as the critical point with statistical significance. (**A**–**C**) The association between the FN1 expression and the NB INSS stage (**A**), likelihood of being high-risk (**B**) and likelihood of a death event (**C**). (**D**–**F**). The association between the PIK3R5 expression and the NB INSS stage (**D**), likelihood of being high-risk (**E)** and likelihood of a death event (**F**). (**G**–**I**) The association between the LPAR6 expression and the NB INSS stage (**G**), likelihood of being high-risk (**H**) and likelihood of a death event (**I**).

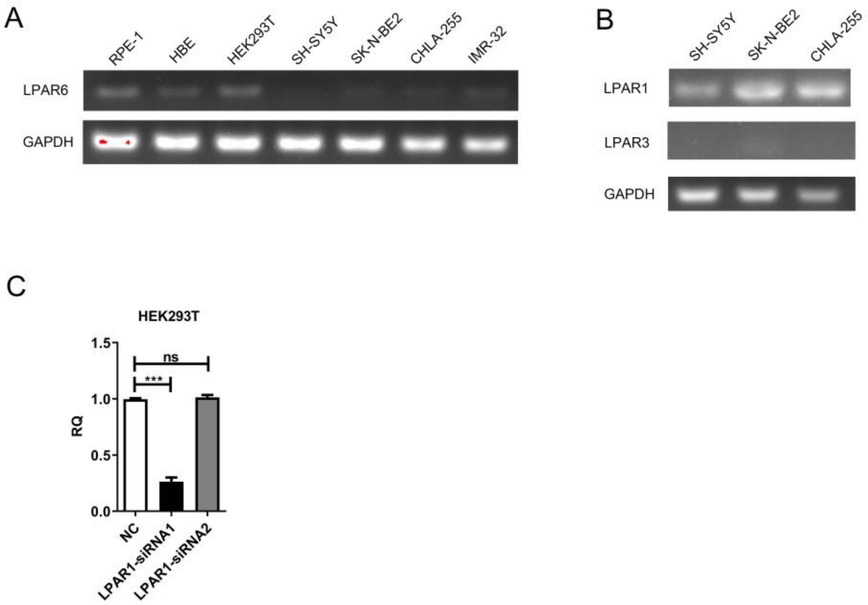

Figure S2. The expression of LPAR1, LPAR3 and LPAR6 in NB cell lines and non-malignant cell lines. (**A**–**B**) The LAPR6 (**A**), LPAR1 and LPAR3 (**B**) expression were detected by PCR. (C) The knockdown efficiency of LPAR1 siRNAs was detected in HEK293T cells by real-time PCR.
